# Web-Based Cognitive Bias Modification Interventions for Psychiatric Disorders: Scoping Review

**DOI:** 10.2196/11841

**Published:** 2019-10-24

**Authors:** Melvyn Zhang, Jiangbo Ying, Guo Song, Daniel S S Fung, Helen Smith

**Affiliations:** 1 National Addictions Management Service Institute of Mental Health Singapore Singapore; 2 Department of Developmental Psychiatry Institute of Mental Health Singapore Singapore; 3 Family Medicine and Primary Care Lee Kong Chian School of Medicine Nanyang Technological University Singapore Singapore Singapore

**Keywords:** cognitive bias, attention bias, psychiatry, eHealth

## Abstract

**Background:**

Cognitive biases refer to automatic attentional or interpretational tendencies, which result in individuals with addictive disorders to automatically attend to substance-related stimuli and those with anxiety disorders to attend to threatening stimuli. To date, several studies have examined the efficacy of cognitive bias modification, and meta-analytical studies have synthesized the evidence for overall efficacy. The clinical utility of cognitive bias modification interventions has previously been limited to the confines of a laboratory, but recent advances in Web technologies can change this.

**Objective:**

This scoping review aimed to determine the scope of Web-based cognitive bias interventions and highlight their effectiveness.

**Methods:**

Databases (PubMed and MEDLINE, EMBASE, PsycINFO, ScienceDirect, and Cochrane Central) were searched from inception to December 5, 2017. The following search terminologies were used: (“attention bias” OR “cognitive bias” OR “approach bias” OR “avoidance bias” OR “interpretative bias”) AND (“Internet” OR “Web” OR “Online”). The methods for this scoping review are based on the previously published protocol. For the synthesis of the evidence, a narrative synthesis was undertaken, as a meta-analysis was not appropriate, given the lack of reported effect sizes and the heterogeneity in the outcomes reported.

**Results:**

Of the 2674 unique articles identified, we identified 22 randomized controlled studies that met our inclusion criteria: alcohol use disorder (n=2), tobacco use disorder (n=2), depressive disorder (n=3), anxiety and depressive symptoms in adolescents (n=3), obsessive-compulsive disorder (OCD; n=2), social anxiety disorder (n=9), and anxiety disorder (n=1). The sample sizes of these studies ranged from 16 to 434 participants. There is preliminary evidence to suggest that Web-based interventions could reduce biases among adolescents with heightened symptoms of anxiety and depression and among individuals with OCD.

**Conclusions:**

This is the first scoping review that mapped out the scope of cognitive bias modification interventions for psychiatric disorders. Web-based interventions have been applied predominantly for social anxiety and addictive disorders. Larger cohorts must be used in future studies to better determine the effectiveness of Web-based cognitive bias interventions.

**International Registered Report Identifier (IRRID):**

RR1-10.2196/10427

## Introduction

### Background

Cognitive biases include automatic, attentional approach tendencies and interpretational tendencies [1]. These automatic processes are believed to be implicated in psychiatric disorders such as addictive disorders and social anxiety disorders [2-4]. Cognitive biases cause individuals with addictive disorders to automatically attend to substance-related stimuli in their environment and those with social anxiety disorders to attend to threatening stimuli. The dual-process theoretical model suggests that these automatic processes arise as a result of increased processing of automatic stimuli, with a corresponding inhibition in normal cognitive control processes [5]. For individuals afflicted with anxiety disorders, when presented with an ambiguous stimulus, anxious individuals tend to make threatening interpretations of the stimuli presented, whereas nonanxious individuals tend to make more positive or benign interpretations [6].

The initial evidence for cognitive biases emerged from experimental psychology, and further research has demonstrated that such biases are amenable to modification. Targeting these automatic biases is important, as conventional psychological therapies, such as cognitive behavioral therapy, typically target only the conscious cognitive control processes and not these underlying automatic biases. Attentional biases can be retrained using either the dot-probe or visual-probe task. These tasks involve pairing the probe (either an asterisk or arrow) with the neutral word or the neutral image all the time [7]. In the visual-probe task, participants are typically presented with a fixation cross at the center of the computer screen, followed by a pair of images (one related to the threatening or triggering stimulus and the other a neutral stimulus, both of which are similar in complexity). Both the stimuli would then disappear, and a probe will appear on the screen. Following the disappearance of the probe, participants are required to indicate the position of the probe on the screen as quickly as possible. The repeated pairing of the probe with the neutral word or neutral image thus facilitates a shift in the attentional focus. Approach biases are retrained using the approach/avoidance task (AAT) [8]. In the AAT, participants are required to either push or pull the images, irrespective of the nature of the stimulus [8]. Interpretative bias modification involves training participants to make positive interpretations through ambiguous scenarios or the word sentence association task [9]. Typically, in the ambiguous scenarios or the word sentence association task, participants are presented with descriptions of a scenario that is ambiguous in terms of emotional valence. Following the disappearance of the ambiguous scenario, participants are presented with a word fragment that would disambiguate the scenario in an anxiety-irrelevant way [6]. Other cognitive bias modification tasks commonly used also include the modified Stroop task and the visual search bias modification task. In the modified Stroop task, participants are presented with both threatening and neutral words, in varying colors, and participants are then asked to name the color of the words while ignoring the semantic content of the word [10]. In the visual search task, participants are asked to identify a target stimulus among a series of distracting stimulus.

To date, several studies have examined the efficacy of cognitive bias modification, and meta-analytical studies have synthesized the evidence for overall efficacy. For substance addictions, Cristea et al [11] reviewed 25 randomized trials (18 for alcohol use disorders and 7 for smoking use disorders) and concluded that bias modification was effective with an effect size of 0.60 (Hedges G). However, they reported no effects of bias modification on other addiction outcomes or on craving [11]. A commentary published [12] in response to this meta-analysis [11] highlighted that a mixture of clinical and nonclinical studies has been included in the evidence synthesis and that, if only clinical studies were considered, the qualitative synthesis demonstrated that there was a significant effect of bias modification. For anxiety and depressive disorders, there remain to be small effect sizes (Hedges G of 0.37) for bias modification based on a previous evaluation of 49 trials [13]. Subsequently, Jones et al [1] in their review of meta-analyses for bias modification reported that attention bias and cognitive bias modification for interpretation did modify biases, with an effect size of 0.24 to 1.16 and 0.52 to 0.81, respectively. There was more evidence for bias modification for anxiety symptoms when compared with depressive symptoms [1]. In addition, Jones et al [1] concluded that both attention bias and cognitive bias modification for interpretations were more effective when delivered in the confines of the laboratory. A laboratory setting enables greater supervision and resultant compliance with the task, given the repetitiveness and monotonous nature of the intervention.

The clinical utility of cognitive bias modification interventions has previously been limited to the confines of a laboratory, but the recent advances in Web technologies can change this. Electronic health, which refers to the process by which health processes and health care are communicated and transferred by an electronic medium and includes Web-based interventions, telephone-delivered therapy, and short message service text messaging, has facilitated this transformation [14]. Web-based therapies also allowed therapy to be delivered across geographical locations and at any time. This new technology is used to deliver conventional therapies, such as cognitive behavioral therapy, as well as cognitive bias modification. To date, there have since been several studies published that have evaluated the effectiveness of Web-based cognitive bias modification. Wittekind et al [15] have previously reported how a Web-based AAT reduced cigarette consumption, cigarette dependence, and compulsive drive among individuals who smoke. Similarly, Blackwell et al [16] used Web technologies for the delivery of cognitive bias modification targeting imagery and interpretation and reported that bias modification was effective in reducing anhedonia symptoms among individuals who were depressed.

### Objectives

Thus, although there have been more Web-based cognitive bias modification interventions, there remains no review that has scoped out the disorders that such interventions target and the effectiveness of these interventions. Given this, the primary objective of this scoping review was to determine the areas in which Web-based cognitive bias modification has been applied. The secondary objective was to synthesize the effectiveness of these interventions and to determine the change in symptoms for the individual psychiatric disorders following bias modification.

## Methods

### Overview

The methods of this scoping review were based on the previously published review protocol [17]. Articles were identified using a search through the following databases: PubMed and MEDLINE, EMBASE, PsycINFO, ScienceDirect, and Cochrane Central. The following search terminologies were used: (“attention bias” OR “cognitive bias” or “approach bias” or “avoidance bias” or “interpretative bias”) AND (“Internet” OR “Web” OR “Online”). The search strategy was modified in accordance to suit the different databases. The databases were searched from inception to December 5, 2017. The search terminology “mobile devices” was not included in our search strategy, as the intent of our search was to identify Web-based interventions. We have previously published another review that has reviewed all the published mobile-based interventions [18].

### Inclusion and Exclusion Criteria

Only articles in the English language were included. Articles were included if (1) the condition examined was a psychiatric disorder, (2) the diagnosis confirmed either using a structured clinical interview or a questionnaire, and (3) cognitive bias modification was delivered through a Web-based modality. Articles were excluded if (1) the intervention failed to include a validated measure for attention bias or cognitive bias, (2) the intervention was delivered using a mobile device or delivered in the form of a game, and (3) cognitive bias modification was part of a pharmacological trial.

### Conditions or Domains Studied

This scoping review was limited to the exploration of Web-based cognitive bias modification for psychiatric disorders.

### Participants

Adult, children, and adolescent populations are included in this review. There were no restrictions on the participants who are included in these studies.

### Intervention and Exposure

The intervention that has been examined is a Web-based cognitive bias modification task. The tasks included are Stroop task, visual-probe/dot-probe task, cognitive bias modification for interpretations, and the visual search task.

### Comparator

Participants are compared with individuals who have received either a placebo or sham training intervention.

### Outcome

The outcome was whether there were changes in biases following the cognitive bias modification intervention.

### Selection of Articles

Articles were deidentified before data extraction. Selection of the relevant publications was conducted independently by 2 authors (MWBZ and JBY). First, articles were screened based on their titles and abstracts. The shortlisted articles were evaluated against the inclusion and exclusion criteria. Disagreements between the 2 authors were resolved through a discussion with a third author (GS). An electronic form was used to record the reasons for the inclusion and the exclusion of the articles.

### Data Extraction

The following information was extracted from each of the articles: (1) publication details (names of the authors and the year of publication), (2) the sample size in the studies, (3) the number of participants in each of the allocated intervention arms (for a randomized trial), (4) study design (cross-sectional, case-controlled, or randomized controlled trial), (5) psychiatric diagnosis of participants, (6) cognitive bias task used, and (7) outcomes of cognitive bias modification (primary outcomes refer to whether biases are present and could be subjected to modification and secondary outcomes refer to other clinical outcomes). The extracted data were cross-checked by another author (JBY) and recorded on a standardized electronic data collation form.

### Statistical Analysis

A narrative synthesis of the effectiveness of Web-based cognitive bias modification for each of the different psychiatric disorders was performed.

## Results

### Findings

**Figure 1:**
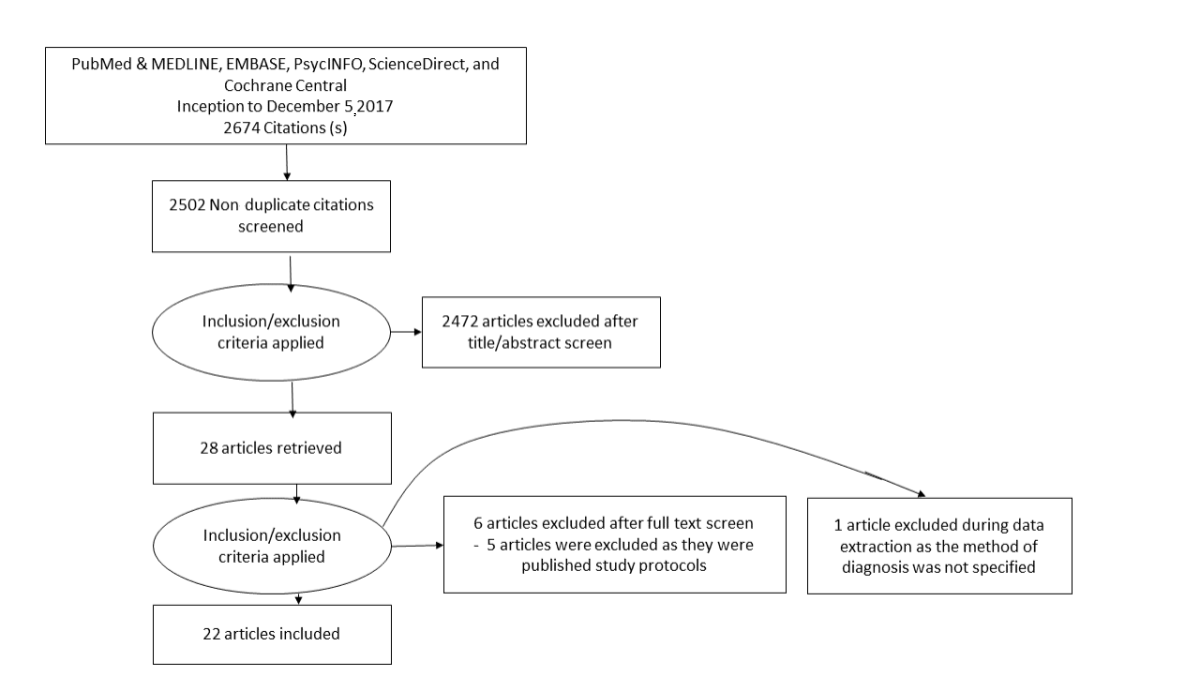
Overview of selection of studies.

A total of 2674 unique articles were identified across all the databases using the predefined search strategy. Of 2674 articles, 172 duplicated articles were removed. On screening of the titles and the abstracts, 2472 papers were excluded as they were not relevant. The full texts of 30 papers were downloaded and screened against the inclusion and exclusion criteria. Moreover, 5 papers were excluded, as they were published protocols, and 1 additional paper was excluded, as it did not specify the method of ascertaining the psychiatric diagnosis. A total of 22 papers were included in this review, which was a combination of both pilot and randomized controlled trials. The sample sizes of these studies ranged from 16 to 434 participants. Figure 1 provides an overview of the selection process of the articles. Multimedia Appendix 1 provides an overview of the characteristics of the included articles [9,15-37].

### Scope of Web-Based Cognitive Bias Modification Interventions

Web-based cognitive bias modification has been evaluated for alcohol use disorder (2 studies), tobacco use disorder (2 studies), depressive disorder (3 studies), anxiety and depressive symptoms in adolescents (3 studies), obsessive-compulsive disorder (OCD; 2 studies), social anxiety disorder (9 studies), and anxiety disorder (1 study).

### Alcohol and Tobacco Use Disorders (4 Studies)

A total of 4 studies evaluated bias modification for addictive disorders. All identified studies recruited their participants by Web mechanisms (Web-based advertisement, websites, and forums), except for the study by Cougle et al [19], in which participants were recruited using doctors’ referral and from the local community (by means of advertisements). Across all the 4 studies, the mean ages of participants were in the 40s with a predominance of females in 3 studies [15,19,20]. In addition, 2 studies included participants with alcohol use disorders [19,21] and 2 included participants with tobacco use disorder [15,20]. Only Cougle et al [19] reported the use of the Diagnostic and Statistical Manual of Mental Disorders, Fifth Edition (DSM-5), diagnostic criteria in ascertaining the diagnosis for the participants, whereas Wittekind et al [15] and Elfeddali [20] reported the use of questionnaires for diagnosis. Wiers et al [21] reported using a questionnaire and self-report for diagnosis. Two studies used the AAT [15,21], and the 2 other studies used the attention bias modification task [19] and interpretative bias modification task [19,20].

Furthermore, 2 studies for alcohol use disorder [19,21], which involved 58 and 136 participants, did not suggest that there was a clear benefit of cognitive bias modification. Wiers et al [21] and Cougle et al [19] reported a reduction in alcohol consumption in intervention and control groups (sham controls and healthy video controls), thus providing no evidence to support bias retraining for reducing alcohol consumption. However, Cougle et al [19] reported that bias modification led to reductions in trait anger and hostility.

Wittekind et al [15] and Elfeddali et al [20] provided preliminary evidence for the effectiveness of the approach and avoidance modification task in smoking. Wittekind et al [15], in their study that included 257 participants reported a reduction in the number of cigarettes smoked and smoking compulsion in participants, whereas Elfeddali et al [20] who in their study included 434 participants reported that bias modification was not effective in continued abstinence.

### Depressive Disorders (3 Studies)

A total of 3 studies [22-24] evaluated bias modification for depressive disorders, and 1 study [22] evaluated cognitive bias modification in conjunction with standard internet-based cognitive behavioral therapy. Of 3 studies, 2 recruited participants from the university and the community, and 1 study [22] recruited participants through a research unit. The participants of all 3 studies were predominantly females, but there was heterogeneity in the mean ages. Moreover, 2 studies [16,22] ascertained diagnosis using the diagnostic interview and Diagnostic and Statistical Manual of Mental Disorders, Fourth Edition, Text Revision (DSM-IV-TR) diagnostic criteria, whereas a study by Pictet et al [23] ascertained the diagnosis of participants using a questionnaire. All studies used cognitive bias modification for interpretation.

Of 3 studies, 2 (which involved 69 and 101 individuals) suggested that there was a change in interpretative biases [22,23], with effect sizes of 0.62 to 2.40 and 0.86, respectively. Anhedonia, which refers to the lack of interest, was a secondary outcome assessed in 2 studies [16,23], but only Pictet et al [23] found a significant reduction in the overall anhedonia scores.

### Anxiety and Depressive Symptoms in Adolescents (3 Studies)

A total of 3 studies examined bias modification for mixed anxiety and depressive symptoms in adolescents. All the studies included secondary school students from the Netherlands. There was a predominance of female participants with mean ages between 14 and 15 years. All studies confirmed the diagnosis of anxiety or depressive symptoms using the Screen for Child Anxiety-Related Emotional Disorders and the Child’s Depression Inventory. For bias modification, 1 study used cognitive bias modification for interpretative bias [24], and the remaining 2 studies [25,26] used attentional visual search.

De Voogd et al [27] reported that attentional bias was significantly reduced in groups that received visual search bias modification. The group size comprised 38 individuals. There was also enhanced attention for positive information following training [26]. Training effects (reduction in biases) were greater for participants who have completed more training sessions. Only 2 of the 3 studies suggested that there was preliminary evidence of a reduction in symptoms of anxiety and depression following training.

### Anxiety Disorders

#### Panic Disorders With or Without Agoraphobia, Social Anxiety Disorders, Posttraumatic Stress Disorder, and Generalized Anxiety Disorders (1 Study)

One study [28], involving 47 participants (24 allocated to intervention and 23 allocated to control), examined bias modification in individuals with a range of anxiety conditions. Individuals sampled were from an anxiety center or medical center. The most common anxiety condition was that of panic disorder, with or without agoraphobia, followed by generalized anxiety disorders and posttraumatic stress disorder. Diagnoses were ascertained using the structured clinical interview for the DSM-IV. Those who (24 participants) received the intervention tended to make more positive interpretations. For the secondary outcome of anxiety scores, both groups (intervention and control) had a reduction.

#### Obsessive-Compulsive Disorders (2 Studies)

Two studies examined bias modification in participants with OCDs. Salemink et al [29] recruited adolescent participants (mean age range 15.1-15.6 years) from a treatment facility, whereas Weil et al [30] recruited participants who had a past diagnosis of OCDs. The sample size in Salemink et al was 16 participants, whereas the sample size in Weil et al was 101 participants. In a study by Salemink et al [29], participants were males, and in a study by Weil et al [30], most participants were females. For the diagnosis of OCDs, it was ascertained by the DSM-IV-TR in a study by Salemink et al [29] and using questionnaires in a study by Weil et al [30]. Cognitive bias modification for interpretation and the AAT was used for bias intervention, among the relatively small sample of individuals. Cognitive bias modification for interpretations reduced the speed with which individuals made OCD-related interpretations. In both studies, there was a reduction of symptoms related to OCD and distress caused by OCD symptoms following the intervention.

#### Social Anxiety Disorders (9 Studies)

Nine studies evaluated bias modification in participants with social anxiety disorders. Of 9 studies, 7 recruited participants through advertisements or Web mechanisms. Sportel et al [31] and de Hullu et al [25] recruited adolescents from regular secondary schools. All the studies included mostly female participants. Eight studies ascertained the diagnosis of social anxiety disorders by means of a clinical interview and 1 study [9] by means of a questionnaire. Stroop task was used in 1 study, visual-probe or dot-probe task in 5 studies, and cognitive bias modification for interpretations in 3 studies.

Biases were found to be present in 7 studies [33,35-37]. For bias modification, 4 studies provided preliminary evidence that bias modification was not effective [31,33,35,37]. Although Boettcher et al [33] reported no changes in biases, the authors did report that there was a significant improvement in social anxiety symptoms following the intervention. Similarly, Neubauer et al [36] also reported a small, although significant, reduction in social anxiety symptoms, despite there being no changes in overall biases. Sportel et al [31] and Brettschnieder et al [34] reported that bias modification was effective, and there was a corresponding reduction in secondary outcome measures (anxiety).

## Discussion

### Principal Findings

This is the first scoping review that maps out the areas in which Web-based cognitive bias modification has been applied and the accompanying findings. Our findings demonstrate that Web technologies have been widely applied for cognitive bias modification for several psychiatric disorders, such as alcohol disorder and social anxiety disorder. Most of the published studies have examined the utility of Web-based cognitive bias modification for social anxiety disorders (9 studies) and addictive disorders (4 studies). Studies involving adolescents with heightened symptoms of anxiety and depression and individuals with OCDs reported positive findings. For depressive disorders, addiction disorders, and social anxiety disorders, there were both positive and negative studies.

One of the key findings from this review is that Web-based cognitive bias interventions have been evaluated among individuals with a diverse range of psychiatric disorders but predominantly for anxiety disorders. The increase in the number of studies conducted evaluating Web technologies in the delivery of cognitive bias modification is expected, given the inherent advantages of using Web technologies. Unlike conventional cognitive bias interventions, Web interventions do not need a therapist, and this enables the intervention to be disseminated to multiple clients [19]. Web technologies also remove geographical barriers that might limit participants from receiving bias interventions [34]. More importantly, Web technologies enable participants to receive the intervention beyond the confines of a laboratory [16], perhaps in the comfort of their own home. As these benefits become more widely recognized, we can expect more studies examining Web-based cognitive bias interventions, together with studies capitalizing on other modalities, such as mobile technologies. Mobile technologies have additional advantages, as they do not require individuals to be connected to the internet to receive the intervention and that it also facilitates individual engaging in training tasks in high-risk situations.

Although the use of Web technologies for the delivery of cognitive bias interventions for psychiatric disorders appears promising, we found that there are studies reporting negative results for depressive disorders, addictive disorders, and social anxiety disorders. Our findings are consistent with Calbring et al and Neubauer et al [35,36] in their head-to-head trials. They reported fewer positive results for Web interventions compared with interventions conducted in the confines of the laboratory. There are several reasons as to why Web cognitive bias modification is less effective in comparison with laboratory-delivered interventions. Studies have reported Web bias modification to be less effective for individuals with social anxiety disorders, as the arousal levels of participants are lesser compared with when the intervention is administered in the laboratory environment, given that in the laboratory environment, participants who are socially anxious are required to interact with others [35,36]. In addition, participants who undertake a Web intervention are more likely to be distracted (such as being disturbed by others) during bias retraining [35]. There is less control over a Web intervention compared with a laboratory-based intervention. De Voogd et al [27] reported that in their study, participants did not adhere to the timelines for training, with some participants failing to undertake the intervention for some days and other participants condensing the training sessions into a few days. Some studies may have failed to have demonstrated significant results because of reduced power with participant attrition [27]. The lack of a positive finding might be also be attributed to the poor motivation when training on the Web for highly repetitive tasks. The lack of a positive finding might also be because of the small sample sizes included in some of these studies. The inclusion of a small sample might have affected the ability to detect any meaningful statistically significant result. The negative findings should not deter future research, examining the potential of Web bias modification interventions, as there remain studies demonstrating positive findings of Web interventions and there remain multiple advantages of a Web approach. Future studies should consider the limitations of existing published studies, as mentioned above. Gamification technologies could be considered to minimize the repetitiveness of tasks, and there have since been studies [38] reporting increased motivation following the incorporation of gamification features. Elements of motivational support could be included in training tasks to minimize attrition and improve compliance to tasks.

In addition, from our scoping review, it seemed that in some studies, a reduction in cognitive biases is associated with an improvement in other psychiatric outcomes (eg, a reduction in interpretative biases is associated with reduced anxiety), but such a finding is not consistent across the studies. This implies that although there might be a reduction in overall biases, it might not directly translate to an improvement in clinical symptoms. In a review of cognitive bias modification for substance use disorders by Cristea et al [11], such inconsistencies have been previously highlighted. In their previous review, they found cognitive bias modification to be moderately effective, with an effect size of 0.60 (Hedges G). However, they did not find that cognitive bias modification helped in improving any of the other secondary outcomes, such as cravings. It might be possible that the change in biases does not immediately result in an improvement in clinical symptoms, and that more time might be needed [11]. There being a positive change in cognitive biases could also possibly be accounted for by participants getting better at the task, as the task used for bias retraining and assessment is the same [11]. Given this, it is important for future research to consider a longer follow-up interval to determine if the changes in biases would result in clinically significant changes in symptoms. Future research should also consider the methods used for assessment and modification, for example, using the visual-probe task for bias retraining, and the modified Stroop task for bias assessment.

This scoping review has several strengths. Our scoping review has helped to bridge the existing gap in the literature. From our review, we found that cognitive bias modification has preliminary effectiveness among adolescents with heightened symptoms of anxiety and depression and individuals with OCDs. These findings are promising, and there should be future adequately powered trials to better evaluate the effectiveness. Our review also highlights there being a need to further evaluate cognitive bias modification among other anxiety disorders, such as posttraumatic stress disorder and possibly that of psychotic disorders. We have undertaken a comprehensive review of the literature for Web cognitive bias interventions for a diverse range of psychiatric disorders, searching through several databases, which captured a proportion, if not all the published studies to date. We have based our scoping review on an a priori review protocol. We have specified the terminologies we have used for the search strategy and applied strict inclusion and exclusion criteria for the selection of the articles identified from the published literature. We have also used standardized data extraction forms. There remain several limitations to this review. As this review was intended to be a scoping review, we did not adhere to the guidelines for a systematic review. Although we have prepared a review protocol and published the protocol, we have not registered this review with PROSPERO. We have not undertaken any form of quality assessment or critical appraisal; hence, we were not able to determine the quality of the studies. We are unable to perform any concrete synthesis of our results, as we have not determined the quality of the studies, and we have included a mixture of randomized trials and pilot randomized trials. The evidence from pilot randomized trials might be misleading because of the small sample sizes.

### Conclusions

This is the first scoping review that has mapped out the scope of cognitive bias modification interventions for psychiatric disorders and their initial findings. Web-based interventions have been predominantly applied for social anxiety and addictive disorders. Larger cohorts must be used in future studies to better determine the effectiveness of Web-based cognitive bias interventions.

## Appendix

Multimedia Appendix 1 Characteristics of the included studies.

## Abbreviations

AAT: approach and avoidance task
DSM-5: Diagnostic and Statistical Manual of Mental Disorders, Fifth Edition
DSM-IV-TR: Statistical Manual of Mental Disorders, Fourth Edition, Text Revision
OCD: obsessive-compulsive disorder

## References

[1] Jones EB, Sharpe L (2017). Cognitive bias modification: a review of meta-analyses. J Affect Disord.

[2] Field M, Cox WM (2008). Attentional bias in addictive behaviors: a review of its development, causes, and consequences. Drug Alcohol Depend.

[3] Cox WM, Fadardi JS, Intriligator JM, Klinger E (2014). Attentional bias modification for addictive behaviors: clinical implications. CNS Spectr.

[4] Heeren A, Mogoașe C, Philippot P, McNally RJ (2015). Attention bias modification for social anxiety: a systematic review and meta-analysis. Clin Psychol Rev.

[5] Stacy AW, Wiers RW (2010). Implicit cognition and addiction: a tool for explaining paradoxical behavior. Annu Rev Clin Psychol.

[6] Otkhmezuri B, Boffo M, Siriaraya P, Matsangidou M, Wiers RW, Mackintosh B, Ang CS, Salemink E (2019). Believing is seeing: a proof-of-concept semiexperimental study on using mobile virtual reality to boost the effects of interpretation bias modification for anxiety. JMIR Ment Health.

[7] Christiansen P, Mansfield R, Duckworth J, Field M, Jones A (2015). Internal reliability of the alcohol-related visual probe task is increased by utilising personalised stimuli and eye-tracking. Drug Alcohol Depend.

[8] Eberl C, Wiers RW, Pawelczack S, Rinck M, Becker ES, Lindenmeyer J (2013). Approach bias modification in alcohol dependence: do clinical effects replicate and for whom does it work best?. Dev Cogn Neurosci.

[9] Steinman SA, Teachman BA (2015). Training less threatening interpretations over the internet: does the number of missing letters matter?. J Behav Ther Exp Psychiatry.

[10] Cisler JM, Bacon AK, Williams NL (2007). Phenomenological characteristics of attentional biases towards threat: a critical review. Cognit Ther Res.

[11] Cristea IA, Kok RN, Cuijpers P (2016). The effectiveness of cognitive bias modification interventions for substance addictions: a meta-analysis. PLoS One.

[12] Wiers R (2016). PLOS | Public Library of Science.

[13] Cristea IA, Kok RN, Cuijpers P (2018). Efficacy of cognitive bias modification interventions in anxiety and depression: meta-analysis. Br J Psychiatry.

[14] Zhang MW, Ho RC (2015). Enabling psychiatrists to explore the full potential of e-health. Front Psychiatry.

[15] Wittekind CE, Feist A, Schneider BC, Moritz S, Fritzsche A (2015). The approach-avoidance task as an online intervention in cigarette smoking: a pilot study. J Behav Ther Exp Psychiatry.

[16] Blackwell SE, Browning M, Mathews A, Pictet A, Welch J, Davies J, Watson P, Geddes JR, Holmes EA (2015). Positive imagery-based cognitive bias modification as a web-based treatment tool for depressed adults. Clin Psychol Sci.

[17] Zhang M, Ying J, Song G, Fung DS, Smith H (2018). Web-based cognitive bias intervention for psychiatric disorders: protocol for a systematic review. JMIR Res Protoc.

[18] Zhang M, Ying J, Song G, Fung DS, Smith H (2018). Attention and cognitive bias modification apps: review of the literature and of commercially available apps. JMIR Mhealth Uhealth.

[19] Cougle JR, Summers BJ, Allan NP, Dillon KH, Smith HL, Okey SA, Harvey AM (2017). Hostile interpretation training for individuals with alcohol use disorder and elevated trait anger: a controlled trial of a web-based intervention. Behav Res Ther.

[20] Elfeddali I, de Vries H, Bolman C, Pronk T, Wiers RW (2016). A randomized controlled trial of web-based attentional bias modification to help smokers quit. Health Psychol.

[21] Wiers RW, Houben K, Fadardi JS, van Beek P, Rhemtulla M, Cox WM (2015). Alcohol cognitive bias modification training for problem drinkers over the web. Addict Behav.

[22] Williams AD, Blackwell SE, Mackenzie A, Holmes EA, Andrews G (2013). Combining imagination and reason in the treatment of depression: a randomized controlled trial of internet-based cognitive-bias modification and internet-CBT for depression. J Consult Clin Psychol.

[23] Pictet A, Jermann F, Ceschi G (2016). When less could be more: investigating the effects of a brief internet-based imagery cognitive bias modification intervention in depression. Behav Res Ther.

[24] de Voogd EL, de Hullu E, Heyes SB, Blackwell SE, Wiers RW, Salemink E (2017). Imagine the bright side of life: a randomized controlled trial of two types of interpretation bias modification procedure targeting adolescent anxiety and depression. PLoS One.

[25] de Hullu E, Sportel BE, Nauta MH, de Jong PJ (2017). Cognitive bias modification and CBT as early interventions for adolescent social and test anxiety: two-year follow-up of a randomized controlled trial. J Behav Ther Exp Psychiatry.

[26] de Voogd EL, Wiers RW, Prins PJ, de Jong PJ, Boendermaker WJ, Zwitser RJ, Salemink E (2016). Online attentional bias modification training targeting anxiety and depression in unselected adolescents: short- and long-term effects of a randomized controlled trial. Behav Res Ther.

[27] De Voogd EL, Wiers RW, Salemink E (2017). Online visual search attentional bias modification for adolescents with heightened anxiety and depressive symptoms: a randomized controlled trial. Behav Res Ther.

[28] Salemink E, Kindt M, Rienties H, van den Hout M (2014). Internet-based cognitive bias modification of interpretations in patients with anxiety disorders: a randomised controlled trial. J Behav Ther Exp Psychiatry.

[29] Salemink E, Wolters L, de Haan E (2015). Augmentation of treatment as usual with online cognitive bias modification of interpretation training in adolescents with obsessive compulsive disorder: a pilot study. J Behav Ther Exp Psychiatry.

[30] Weil R, Feist A, Moritz S, Wittekind CE (2017). Approaching contamination-related stimuli with an implicit Approach-Avoidance Task: can it reduce OCD symptoms? An online pilot study. J Behav Ther Exp Psychiatry.

[31] Sportel BE, de Hullu E, de Jong PJ, Nauta MH (2013). Cognitive bias modification versus CBT in reducing adolescent social anxiety: a randomized controlled trial. PLoS One.

[32] Andersson G, Westöö J, Johansson L, Carlbring P (2007). Cognitive bias via the internet: a comparison of web‐based and standard emotional stroop tasks in social phobia. Cogn Behav Ther.

[33] Boettcher J, Leek L, Matson L, Holmes EA, Browning M, MacLeod C, Andersson G, Carlbring P (2013). Internet-based attention bias modification for social anxiety: a randomised controlled comparison of training towards negative and training towards positive cues. PLoS One.

[34] Brettschneider M, Neumann P, Berger T, Renneberg B, Boettcher J (2015). Internet-based interpretation bias modification for social anxiety: a pilot study. J Behav Ther Exp Psychiatry.

[35] Carlbring P, Apelstrand M, Sehlin H, Amir N, Rousseau A, Hofmann SG, Andersson G (2012). Internet-delivered attention bias modification training in individuals with social anxiety disorder - a double blind randomized controlled trial. BMC Psychiatry.

[36] Neubauer K, von Auer M, Murray E, Petermann F, Helbig-Lang S, Gerlach AL (2013). Internet-delivered attention modification training as a treatment for social phobia: a randomized controlled trial. Behav Res Ther.

[37] Boettcher J, Hasselrot J, Sund E, Andersson G, Carlbring P (2013). Combining attention training with internet-based cognitive-behavioural self-help for social anxiety: a randomised controlled trial. Cogn Behav Ther.

[38] Boendermaker WJ, Boffo M, Wiers RW (2015). Exploring elements of fun to motivate youth to do cognitive bias modification. Games Health J.

