# Supplementary material for: Web-Based Cognitive Bias Modification Interventions for Psychiatric Disorders: Scoping Review
Source: JMIR Ment Health. 2019 Oct 24;6(10):e11841. doi: 10.2196/11841 (PMC6915808; doi:10.2196/11841)
Supplement: Multimedia Appendix 1 [file mental_v6i10e11841_app1.pdf]

Table 1. Characteristics of the Included Studies

| Disorders | Study and country of study | Study design | Sample size | Demographics of sample | Mechanism of recruitment | Psychiatric diagnoses | Method of diagnosis | Attention or cognitive bias task | Primary outcomes | Secondary outcomes |
|---|---|---|---|---|---|---|---|---|---|---|
| **Addictive disorders** | | | | | | | | | | |
| | Wiers et al [17]; the Netherlands | Randomized trial with 4 experimental conditions compared against sham training control | 136 problem drinkers: 17 assigned to American Association for the Certification of Training Program, 27 assigned to ATT[a]100 explicit, 35 | Average age of 46.1, 48.1, 46.9, 49.4 and 48.7 years, respectively; gender ratio (females to males): 9:8; 10:27; 14:21; 14:19; 15:9 | Via newspaper and online advertising and a website | Alcohol use disorder | Alcohol Use Disorders Identification Test and alcohol consumption based on the self-reported timeline follow-back procedure | Attention control training and approach bias retraining | Bias present and subjected to manipulation | Reduction in drinking in all intervention arms (attention control training, approach bias retraining, and sham control); no evidence of stronger reduction of alcohol consumption following active training |

| | | | | | | | | | | |
|---|---|---|---|---|---|---|---|---|---|---|
| | | | assigned to ATT100 implicit, 33 assigned to ATT90 implicit, and 24 assigned to ATT50 control | | | | | | | compared with sham training |
| | Wittekind et al [13]; Hamburg, Germany | Randomized trial with 2 experimental conditions (AAT) and a wait-list control | 257 participants, 87 allocated to standard AAT, 85 allocated to modified AAT, and 85 allocated to wait-list control group | Standard AAT: age of 44.30 years, 54 females, 33 males, 24.27 years of smoking, and 20.31 cigarettes per day; modified AAT: age of 43.65 years, 56 females, 29 males, 25.09 years of smoking, and | Invitation to participate posted in several smoking-related internet forums | Tobacco use disorder | Questionnaire (Fagerström Test for Nicotine Dependence and Cigarette Dependence Scale-12) | AAT (only avoidance task used as intervention) | Bias present and subjected to manipulation | Significant reduction in number of cigarettes smoked and compulsion among participants who received intervention versus control; reduction of cigarette dependence and compulsive drive for |

| | | | | | | | | | | | |
|---|---|---|---|---|---|---|---|---|---|---|---|
| | | | | 20.13 cigarettes per day; control group: age of 41.55 years, 47 females, 38 males, 22.62 years of smoking, and 20.52 cigarettes per day | | | | | | smoking most significantly in those assigned to the standard AAT |
| | Elfeddali et al [16]; the Netherlands | Randomized trial involving either 6 sessions of bias modification or placebo | 434 participants, 224 assigned to ABM[b] group and 210 assigned to control group | 68.9% female, average age 40.76 years, average consumption 17.78 cigarettes, 91.7% have had made quit | Online recruitment with advertisement on online international news pages referring to a website | Tobacco use disorder | Questionnaire (FagerstrÖm Test for Nicotine Dependence) | ABM (visual-probe task); approach bias using reaction time paradigms | Significant attentional bias toward smoking-related cues among heavy smokers, whereas bias was not significant in | Nonsignificant results for multiple-session ABM interventions; Web-based, multiple-session ABM intervention was |

| | | | | | | | | | | |
|---|---|---|---|---|---|---|---|---|---|---|
| | | training over 2 weeks | | attempts; ABM group: mean age 40.96 years, 67.4% females, 17.49 cigarettes per day; control group: mean age 40.54 years, 70.5% female, 18.09 cigarettes per day | | | | | light to moderate smokers; significant approach bias for cigarette-related stimuli | not effective in ensuring continued abstinence; ABM training effects did not generalize to that of approach bias |
| | Cougle et al [15]; the United States | Randomized trial involving either 8 Web-based sessions of IBM-H[c] or a HVC[d] | 58 individuals, with 30 receiving IBM-H and 28 receiving HVC | Mean age of sample was 40.71 years, 69.0% female; IBM-H: 70% females, mean age 39.33 years; HVC: 67.9% | Craigslist, Reddit, bus advertisement, doctor referrals, and flyers posted around the local community | Alcohol use disorder with elevated trait anger | Endorsement of 4 of the 11 symptoms of DSM-5[e] modified alcohol module of the Mini-International Neuropsychiatric | IBM-H | Greater reduction in interpretative bias in the intervention group compared with the control group | IBM-H lead to greater reduction in trait anger than HVC. IBM-H also led to reductions in anger expression; both groups |

| | | | | | | | | | | |
|---|---|---|---|---|---|---|---|---|---|---|
| | | | | females, mean age 42.18 years | | | Interview; trait anger based on the State-Trait Anger Expression Inventory-2 (STAXI-2) | | | (intervention and control) reported a reduction in alcohol consumption |
| **Depressive disorders** | | | | | | | | | | |
| | William et al [18]; Australia | Randomized trial that evaluates an 11-week intervention (1-week CBM[f]-I/10 weeks ICBT[g]) to that of a wait-list control | 38 participants for intervention arm; 31 participants in wait-list control arm | Intervention: 77% female, mean age 44.28 years; control: 75% female, mean age 45.35 years | Via the research arm of the Clinical Research Unit for Anxiety and Depression | Depressive disorder | Diagnostic interview using the Mini-International Neuropsychiatric Interview Version 5.0.0 | CBM targeting imagery and interpretation bias (ambiguous scenarios test—depression and the scrambled sentence test) | Change in interpretation bias mediated the reduction in depressive symptoms | Significant reduction in depressive symptoms and distress with effect sizes (Cohen *d* of 0.62-2.40) following CBM-I and ICBT intervention; treatment superiority compared with |

| | | | | | | | | | | |
|---|---|---|---|---|---|---|---|---|---|---|
| | | | | | | | | | | wait-list control demonstrated |
| | Pictet et al [19]; Switzerland | Randomized trial evaluating CBM-I delivered online among 3 cohorts (intervention, wait-list control, and control CBM condition) | 101 individuals; with 34 randomized to the imagery CBM condition, control CBM condition (34), and wait-list condition (33) | Imagery CBM: mean age was 27.50 years, 82% female, BDI[h] was 27.56; control CBM: mean age was 27.44 years, 85% female, BDI was 25.94; wait-list: mean age was 25.06 years, 70% female, BDI was 24.52 | Via flyers posted in the local university and advertisements on local websites | Depressive disorder | Questionnaire (scored above 14 on the BDI-II both at screening and baseline assessment) | CBM targeting imagery (ambiguous scenarios test for depression-related bias) | Significant changes in interpretation bias across time and among those who were assigned to the intervention | Depressive symptoms (as measured by BDI-scores) decreased across time compared with those who received the CBM intervention; reduction in anhedonia symptoms in sample that received CBM; Cohen *d* for improvements in depressive symptoms was 0.86 |

| | | | | | | | | | | |
|---|---|---|---|---|---|---|---|---|---|---|
| | Blackwell et al [14]; Oxford, United Kingdom | Randomized trial involving 12 sessions of imagery CBM intervention completed in a home environment by means of the internet | 150 individuals: 76 allocated to the imagery intervention and 74 in the control group | Imagery CBM: 68% female, mean age 37.64 years, baseline BDI score: 29.96; control: 69% female, mean age 33.28 years, baseline BDI score: 31.14 | Via advertisement in local media, websites and community, university, and health settings in the local area | Depressive disorder | DSM-IV-TR[i] criteria for current major depressive episode as assessed by mean of a semi-SCID[j] | Imagery CBM | No significant reduction in negative interpretative bias | No significant change in the depressive scores (BDI-II) from baseline to postintervention; imagery CBM did help to improve anhedonia symptoms; imagery CBM only helped to improve depression symptoms for participants with less than 5 episodes of depression and who managed to engage to a threshold level of imagery |

| | | | | | | | | | |
|---|---|---|---|---|---|---|---|---|---|
| **Anxiety and depressive symptoms in adolescents** | | | | | | | | | |
| | De Voogd et al [20]; The Netherlands | Randomized trial with 3 experimental conditions (text-based scenario training, picture-word imagery training, or neutral control training) | 119 individuals: 36 randomized to scenario training, 44 to picture-word training, and 39 in control group | Whole sample: 63% female, mean age 15.68 years; scenario training: mean age 15.78 years, 63.9% female; picture-word training: mean age 15.76 years, 59.1% female; control group: mean age 15.51 years, 66.7% female | From 4 secondary schools in the Netherlands | Heightened anxiety and depressive symptoms in adolescents | Score of more than 16 on the SCARED and more than 7 on the CDI[l] | CBM (scenario training vs picture-word training); interpretation bias was assessed using scrambled sentence task under cognitive load | Positive interpretation bias on the recognition task pretraining | Decrease in anxiety and depression across all intervention groups; perseverative negative thinking decreased and self-esteem increased across all the conditions |
| | De Voogd et al [23]; the Netherlands | Randomized trial comparing 8 online sessions | 108 individuals: 38 randomized to VS training, 32 | Whole sample: 66.7% female, mean age 14.45 years; | From 4 secondary schools in the Netherlands | Heightened anxiety and depressive | Score of more than 16 on the SCARED and | VS ABM; recognition task for interpretation bias assessment | Significant attentional bias for negative information | Irrespective of training condition, an overall |

| | | | | | | | | | | |
|---|---|---|---|---|---|---|---|---|---|---|
| | | of VS[m] bias modification to placebo training to no training | to VS placebo, and 38 to control | VS training: mean age 14.73 years, 63.2% female; VS placebo: mean age 14.31 years, 65.6% female; control: mean age 14.29 years, 71.1% females | | symptoms in adolescents | more than 7 on the CDI | | present at baseline; attentional bias was significantly reduced in groups assigned to VS bias modification; completion of greater number of sessions was associated with larger reduction in attentional biases; increase in the positive interpretation bias in the VS training group (generalization of bias modification) | reduction in symptoms of anxiety and depression and an increase in emotional resilience were observed up to 6 months later |

| | | | | | | | | | | |
|---|---|---|---|---|---|---|---|---|---|---|
| | De Voogd et al [22]; the Netherlands | Randomized trial with 4 experimental conditions VS training, (DP[n] training, VS or DP control condition) | 340 adolescents with symptoms of anxiety and depression: 126 allocated to VS training, 128 allocated to DP training, 38 allocated to VS placebo, 48 allocated to DP placebo | Whole sample: 57.6% females, mean age 14.41 years; VS training: mean age 14.41 years, 58.7% females; VS placebo: mean age 14.39 years, 63.2% females. DP training: mean age 14.30 years, 56.3% females; DP placebo: mean age 14.66 years, 54.2% females | From 14 regular high schools in the Netherlands | Anxiety and depression in adolescents | Anxiety symptoms were assessed with the SCARED; depressive symptoms were assessed with the CDI | DP and VS attentional training | VS training enhanced attention for positive information. This effect was stronger for participants who completed more training sessions | Symptoms of anxiety and depression reduced, whereas emotional resilience improved. However, these effects were not especially pronounced in the active conditions. |

| | | | | | | | | | | |
|---|---|---|---|---|---|---|---|---|---|---|
| **Anxiety disorders** | | | | | | | | | | |
| | Salemick et al [24]; the Netherlands | Randomized trial with 2 experimental conditions (100% positive interpretations of ambiguous social scenarios or 50% and 50% negative interpretations) | 47 participants: 24 allocated to the positive CBM-I condition and 23 allocated to control condition | Study 1: 14 diagnosed with PD[o] with or without agoraphobia, 8 GAD[p], 6 SAD[q], 6 PTSD[r], and 2 agoraphobia with history of PD; positive CBM group: mean age 41.9 years, 15 females, 14.9 years mean duration of diagnosis; control group: mean age 38.6 years, 11 | Altrecht Academic Anxiety Centre or Mesos Medical Centre | Anxiety disorders (PD with or without agoraphobia, SAD, PTSD, or GAD) | SCID version 1 | Positive CBM for interpretations | Those participants who had received the CBM-I training program interpreted ambiguous information in a more positive manner compared with those who were in the placebo control condition; actual change in biases cannot be determined as there was no preassessment of baseline | Both the intervention and control groups have had a reduction in anxiety, depressive, and general psychological distress symptoms |

| | | | | | | | | | | |
|---|---|---|---|---|---|---|---|---|---|---|
| | | | | females, 15.7 years mean duration of diagnosis | | | | | biases; online training was successful in modifying biases across a range of anxiety disorders | |
| | Anderson et al [28]; Sweden | Randomized trial with 2 experimental arms, Stroop task, or Web Stroop task | 87 individuals, with 44 allocated to Stroop task and 43 to Web Stroop task | 50 females, 45 males: 8 participants were excluded; Stroop task: mean age 36.6 years; Web Stroop task: mean age 39.0 years; total sample: mean age 37.8 years | Via newspaper advertisements and a website for a treatment study of SAD | SAD | Self-reported questionnaires with SCID | Two different versions of the emotional Stroop color-naming task were used—Stroop Task and Web Stroop Task | Individuals allocated to the Stroop task color-named socially threatening words slower than neutral words; individuals allocated to the Web Stroop slower to color name neutral words compared with socially | Web-based Stroop had a facilitation effect, in that individuals with social phobia tend to indicate choices for words related to social threats more quickly |

| | | | | | | | | | | |
|---|---|---|---|---|---|---|---|---|---|---|
| | | | | | | | | | threatening words | |
| | Calbring et al [31]; Sweden | Randomized trial with 2 experimental conditions (active and placebo condition); active condition involves training to direct attention away from the threat | 79 individuals: 40 allocated to the treatment and 39 to the placebo | Treatment group: 65.0% females, mean age 35.1 years, 72.5% generalized SAD, and 27.5% nongeneralized SAD;<br>placebo group: 71.8% females, mean age 38.0 years, 76.9% generalized SAD, 23.1% nongeneralized SAD | By media advertisements | SAD | DSM-IV diagnosis and interview based on the SCID | Probe detection task | Attention bias was not affected by the frequency of the training and by the experimental condition assigned; bias modification via the internet is not effective | No significant differences in all measured outcomes variables |
| | Boettcher et al [29]; Sweden | Randomized trial with 6 | 129 individuals: 22 allocated to | Total sample: 36% male, | By advertisements in regional | SAD | Social Phobia Screening | DP task | Participants did not exhibit an | Individuals in the attend to |

| | | | | | | | | | | |
|---|---|---|---|---|---|---|---|---|---|---|
| | | experimental conditions | “attend to threat words”, 21 allocated to “attend to threat words and faces,” 21 allocated to “attend to positive words,” 22 allocated to “attend to positive words and faces,” 21 allocated to control condition words, 22 allocated to control condition words and faces | mean age 38.26 years; control words: 52% male, mean age 42.81 years; control words + faces: 41% male, mean age 39.32 years; toward negative words: 18% male, mean age 35.50 years; toward negative words + faces: 29% male, mean age 35.57 years; toward positive words: 43% male, mean age | newspaper in Sweden | | Questionnaire; SCID Axis I disorders | | attention bias at baseline; there was no change in attention bias from baseline to after the intervention across all conditions | threat group improved significantly in SAD symptoms from pre- to postassessment |

| | | | | | | | | | | |
|---|---|---|---|---|---|---|---|---|---|---|
| | | | | 38.05 years; toward positive words + faces: 36% male, mean age 38.36 years | | | | | | |
| | Neubauer et al [32]; Germany | Randomized trial with 2 experimental conditions (modified DP paradigm or control condition without attention modification) | 56 individuals: 28 allocated to the intervention and 28 allocated to the control condition | Intervention group: mean age 40.07 years, 57.1% females; control group: mean age: 39.00 years, 75.0% females | By press announcements in local newspapers | SAD | Diagnostic assessment including a clinical interview evaluating DSM-IV criteria for Axis I disorders | DP task | Before the intervention, attention bias (difficulties with disengagement) was found to be present; groups did not differ significantly in disengagement scores on completion of intervention and | Small, although significant, reduction in SAD and depression in both groups |

| | | | | | | | | | | |
|---|---|---|---|---|---|---|---|---|---|---|
| | | | | | | | | | at follow-up (no change in bias) | |
| | Boettcher et al [33]; Sweden | Randomized trial comparing ICBT with attentional training against ICBT with control training | 133 participants (66 allocated to ICBT with attention training and 67 allocated to ICBT with control training) | Total sample: mean age 33.4 years; attention training group: 32% male; control group: 40% male | Via the internet and advertisement in national newspapers | SAD | SCID Axis I disorders | Attention training and control training (based on the DP task) | No change in attention bias from pre- to postassessment; ABM was not effective | Improvement in SAD symptoms from pre- to postassessment |
| | Sportel et al [27]; the Netherlands | Randomized trial with 3 experimental conditions (CBT vs control vs CBM) | 84 individuals randomized to receive cognitive behavioral therapy; 86 individuals randomized to CBM; 70 individuals as control | CBM group: 77% females, mean age 14.12 years; CBT group: 67% females, mean age 14.06 years; control group: 77% females, mean age 14.11 years | Adolescents in the first and second year of regular secondary schools in the Northern part of the Netherlands were invited | SAD | Clinical assessment using the Anxiety Disorders Interview Schedule for Children | Visual-probe Task | Less negative interpretations in the CBM group compared with the control and CBT groups; attentional biases to friendly faces improved in the CBM condition | Individuals in the CBT and CBM conditions achieved reduction in SAD symptoms at 6-month follow-up; at 12-month follow-up, individuals who received CBM had a |

| | | | | | | | | | | |
|---|---|---|---|---|---|---|---|---|---|---|
| | | | | | | | | | | stronger decrease of negative automatic associations |
| | Brettschneider et al [30]; Germany and Switzerland | Cross-sectional study | 39 individuals | 64.1% female, mean age 35.3 years | Through posted announcements in several self-help internet forums | SAD | SCID | CBM for interpretations | Reduction of cognitive processing bias from pre- to postassessment (*d*=0.77) | SAD symptoms decreased from pre- to postassessment and one-third of the participants did not meet the criteria for social phobia. |
| | Steinman et al [8]; the United States | Randomized trial with 5 experimental conditions (control—half of scenarios ended positively and half | 350 individuals with SAD | 92.9% of participants from the United States, age range from 18 to 64 years, mean age 35.44 years; CBM control: | Via the internet and Amazon. Mechanical Turk | SAD | Questionnaire—Social Interaction and Anxiety Scale | CBM for interpretations | Online bias modification could successfully modify interpretations to be more positive or negative relative | No resultant change in other secondary outcomes |

| | | | | | | | | | | |
|---|---|---|---|---|---|---|---|---|---|---|
| | | negatively) or 4 positive conditions (word fragments varied on number of letters missing from 0 to 3) | | 62.50% female, mean age 39.04 years; CBM-0: 61.73%female, mean age 32.38 years; CBM-1: 57.38% female, mean age 32.65 years; CBM-2: 82.81%female, mean age 37.08 years; CBM-3: 60.94%female, mean age 35.70 years | | | | | to control condition | |

| | | | | | | | | | | |
|---|---|---|---|---|---|---|---|---|---|---|
| | de Hullu [21]; the Netherlands | Randomized trial with 2 experimental conditions (assigned to 10-week multifaceted CBM training or 10-week school-based cognitive behavioral group training) | 240 individuals with SAD: 84 assigned to CBT group, 70 to control group, and 86 to CBM group | Total sample mean age 13.6 years | Adolescents who were in the first and second year of regular secondary schools invited | SAD | Clinical interview using the Revised Child Anxiety and Depression Scale; Anxiety Disorders Interview Schedule for Children | CBM for interpretations | CBM was effective in changing interpretative bias | Reduction in self-reported anxiety across all interventional arms |
| | Salemink et al [25]; the Netherlands | Pilot randomized Trial (CBM-I training or placebo condition) | 16 individuals with OCD[s] with 9 allocated to CBM-I training and 7 to placebo variant | CBM-I group: mean age 15.6 years, 5 females, duration of OCD symptoms 6.2 years; placebo control group: mean age 15.1 years, 5 | Adolescents referred for either inpatient or outpatient treatment for OCD at the academic center for child and adolescent psychiatry | OCD | DSM-IV-TR Criteria | CBM for interpretations | CBM-I effective in reducing the speed of making OCD-related immediate online interpretations—training reduced the likelihood and speed of interpreting | Reduction in the OCD symptoms among those who received the intervention |

| | | | | | | | | | | |
|---|---|---|---|---|---|---|---|---|---|---|
| | | | | females, 4.5 years duration of symptoms | | | | | information in OC manner | |
| | Weil et al [26]; Germany | Pilot randomized trial (2 experimental conditions—standard AAT or AAT with lock or wait-list control) | 101 individuals with obsessive-compulsive disorder: 37 allocated to Standard AAT (23 with contamination-related symptoms), 27 allocated with AAT with lock (19 with contamination-related symptoms), and 37 allocated with wait-list control (24 with contamination- | Standard AAT: mean age 40.83 years, 5 males, 21.33 years duration; AAT with lock: mean age 38.53 years, 6 males, and 21.51 years duration; control group: mean age 38.54 years, 5 males, and 18.09 years duration | Via OCD-specific websites and internet forums as well as via a database of former patients with established diagnosis of OCD in Germany | OCD | Questionnaires—OCD symptoms were assessed with the self-rating version of the Yale-Brown Obsessive-Compulsive Scale and the Obsessive-Compulsive Inventory Revised | Approach and avoidance task | Findings tentatively suggest that the AAT might be effective in reducing washing-/contamination-related OCD symptoms | Significant reduction in distress caused by OCD symptoms in the intervention group compared with the control group |

| | | | | | | | | | | |
|---|---|---|---|---|---|---|---|---|---|---|
| | | | related symptoms) | | | | | | | |

[a]AAT: approach/avoidance task.

[b]ABM: attention bias modification.

[c]IBM-H: Interpretative Bias Modification for Hostility.

[d]HVC: healthy video control condition.

[e]DSM-5: Diagnostic and Statistical Manual of Mental Disorders.

[f]CBM: cognitive bias modification.

[g]ICBT: internet-based cognitive behavioral therapy.

[h]BDI: Beck Depression Inventory.

[i]DSM-IV-TR: Diagnostic and Statistical Manual of Mental Disorders, Fourth Edition, Text Revision.

[j]SCID: Semistructured Clinical Interview for DSM-5.

[k]SCARED: Screen for Child Anxiety-Related Emotional Disorders.

[l]CDI: Children's Depression Inventory.

[m]VS: visual search.

[n]DP: dot probe.

[o]PD: panic disorder.

[p]GAD: generalized anxiety disorder.

[q]SAD: social anxiety disorder.

[r]PTSD: posttraumatic stress disorder.

[s]OCD: obsessive-compulsive disorder.

References:

8. Steinman SA, Teachman BA. Training less threatening interpretations over the Internet: Does the number of missing letters matter? J Behav Ther Exp Psychiatry. 2015;49(Pt A):53-60.
13. Wittekind CE, Feist A, Schneider BC, Moritz S, Fritzsche A. The approach-avoidance task as an online intervention in cigarette smoking: a pilot study. J Behav Ther Exp Psychiatry. 2015;46:115-20.
14. Blackwell SE, Browning M, Mathews A, Pictet A, Welch J, Davies J, et al. Positive Imagery-Based Cognitive Bias Modification as a Web-Based Treatment Tool for Depressed Adults: A Randomized Controlled Trial. Clin Psychol Sci. 2015;3(1):91-111.
15. Cougle JR, Summers BJ, Allan NP, Dillon KH, Smith HL, Okey SA, et al. Hostile interpretation training for individuals with alcohol use disorder and elevated trait anger: A controlled trial of a web-based intervention. Behav Res Ther. 2017;99:57-66.
16. Elfeddali I, de Vries H, Bolman C, Pronk T, Wiers RW. A randomized controlled trial of Web-based Attentional Bias Modification to help smokers quit. Health Psychol. 2016;35(8):870-80.
17. Wiers RW, Houben K, Fadardi JS, van Beek P, Rhemtulla M, Cox WM. Alcohol cognitive bias modification training for problem drinkers over the web. Addict Behav. 2015;40:21-6.
18. Williams AD, Blackwell SE, Mackenzie A, Holmes EA, Andrews G. Combining imagination and reason in the treatment of depression: a randomized controlled trial of internet-based cognitive-bias modification and internet-CBT for depression. J Consult Clin Psychol. 2013;81(5):793-9.
19. Pictet A, Jermann F, Ceschi G. When less could be more: Investigating the effects of a brief internet-based imagery cognitive bias modification intervention in depression. Behav Res Ther. 2016;84:45-51.
20. de Voogd EL, de Hullu E, Burnett Heyes S, Blackwell SE, Wiers RW, Salemink E. Imagine the bright side of life: A randomized controlled trial of two types of interpretation bias modification procedure targeting adolescent anxiety and depression. PLoS One. 2017;12(7):e0181147.
21. de Hullu E, Sportel BE, Nauta MH, de Jong PJ. Cognitive bias modification and CBT as early interventions for adolescent social and test anxiety: Two-year follow-up of a randomized controlled trial. J Behav Ther Exp Psychiatry. 2017;55:81-9.
22. de Voogd EL, Wiers RW, Prins PJ, de Jong PJ, Boendermaker WJ, Zwitser RJ, et al. Online attentional bias modification training targeting anxiety and depression in unselected adolescents: Short- and long-term effects of a randomized controlled trial. Behav Res Ther. 2016;87:11-22.
23. De Voogd EL, Wiers RW, Salemink E. Online visual search attentional bias modification for adolescents with heightened anxiety and depressive symptoms: A randomized controlled trial. Behav Res Ther. 2017;92:57-67.
24. Salemink E, Kindt M, Rienties H, van den Hout M. Internet-based cognitive bias modification of interpretations in patients with anxiety disorders: a randomised controlled trial. J Behav Ther Exp Psychiatry. 2014;45(1):186-95.
25. Salemink E, Wolters L, de Haan E. Augmentation of Treatment As Usual with online Cognitive Bias Modification of Interpretation training in adolescents with Obsessive Compulsive Disorder: A pilot study. J Behav Ther Exp Psychiatry. 2015;49(Pt A):112-9.

26. Weil R, Feist A, Moritz S, Wittekind CE. Approaching contamination-related stimuli with an implicit Approach-Avoidance Task: Can it reduce OCD symptoms? An online pilot study. Journal of Behavior Therapy and Experimental Psychiatry. 2017;57:180-8.
27. Sportel BE, de Hullu E, de Jong PJ, Nauta MH. Cognitive bias modification versus CBT in reducing adolescent social anxiety: a randomized controlled trial. PLoS One. 2013;8(5):e64355.
28. Andersson G, Westoo J, Johansson L, Carlbring P. Cognitive bias via the Internet: a comparison of Web-based and standard emotional Stroop tasks in social phobia. Cogn Behav Ther. 2006;35(1):55-62.
29. Boettcher J, Leek L, Matson L, Holmes EA, Browning M, MacLeod C, et al. Internet-based attention bias modification for social anxiety: a randomised controlled comparison of training towards negative and training towards positive cues. PLoS One. 2013;8(9):e71760.
30. Brettschneider M, Neumann P, Berger T, Renneberg B, Boettcher J. Internet-based interpretation bias modification for social anxiety: A pilot study. J Behav Ther Exp Psychiatry. 2015;49(Pt A):21-9.
31. Carlbring P, Apelstrand M, Sehlin H, Amir N, Rousseau A, Hofmann SG, et al. Internet-delivered attention bias modification training in individuals with social anxiety disorder--a double blind randomized controlled trial. BMC Psychiatry. 2012;12:66.
32. Neubauer K, von Auer M, Murray E, Petermann F, Helbig-Lang S, Gerlach AL. Internet-delivered attention modification training as a treatment for social phobia: a randomized controlled trial. Behav Res Ther. 2013;51(2):87-97.
33. Boettcher J, Hasselrot J, Sund E, Andersson G, Carlbring P. Combining attention training with internet-based cognitive-behavioural self-help for social anxiety: a randomised controlled trial. Cogn Behav Ther. 2014;43(1):34-48.
